# Potency, immunogenicity, and efficacy of rabies vaccine: In vitro and in vivo approach

**DOI:** 10.1002/iid3.1198

**Published:** 2024-02-27

**Authors:** Fahima Akter, Md. Shamimuzzaman

**Affiliations:** ^1^ Department of Microbiology and Hygiene Bangladesh Agricultural University Mymensingh Bangladesh; ^2^ Department of Microbiology Jashore University of Science and Technology Jashore Bangladesh; ^3^ Directorate General of Drug Administration Ministry of Health and Family Welfare Dhaka Bangladesh

**Keywords:** FAVN, immunogenicity, in vivo and in vitro, NIH, rabies vaccine, SRID, vaccine potency

## Abstract

**Background:**

Rabies, a potentially lethal virus, affects more than 150 countries. Although the rabies vaccine and immunoglobulin have been available since 1908, Bangladesh is new to vaccine manufacturing. We checked the quality of the local manufacturing rabies vaccine for substandard.

**Methods:**

The potency and immunogenicity of 20 vaccines were analyzed by three in vivo and in vitro methods from March 2020 to May 2023. Single radial immunodiffusion, fluorescent antibody virus neutralization, and national institutes of health tests were carried out to evaluate the vaccine's efficacy to provide sufficient protection against the rabies virus.

**Results:**

The potency of the rabies vaccine was determined by the in vitro SRID method by measuring glycoprotein content. An average of 16 articles from each batch was calculated. The minimum and maximum average mean values of the 20 batches were 5.058 and 5.346, respectively. The variance was calculated at 0.00566. We found a coefficient of variation (CV) between 9.36% and 14.80%. The 100% sample was satisfactory, as these samples had a potency of over 2.5 IU/mL. To observe immunogenicity, we applied the FAVN method for determining antibody titers. An average of 16 articles from every batch were counted to quantify antibody titers. The mean quantity of antibody titers ranged from 2.389 to 3.3875. The CV was slightly lower because of the dispersion of the data. At last, we performed an in vivo method, the NIH test method, to determine potency based on mortality rate. We found a mean value of 4.777 IU/SHD with a standard deviation of 1.13 IU/SHD. All 20 batches were found 100% satisfactory in the NIH test.

**Conclusion:**

The study implies that the rabies human vaccines manufactured in Bangladesh are potent enough to provide sufficient immunogenicity. Our research is warranted testimony for healthcare providers who work to extirpate rabies.

## Graphical abstract

To describe immunogenecity, we checked potency of rabies vaccine. Single radial immunodiffusion method is in vitro, National Institutes of Health and fluorescent antibody virus neutralization is in vivo.

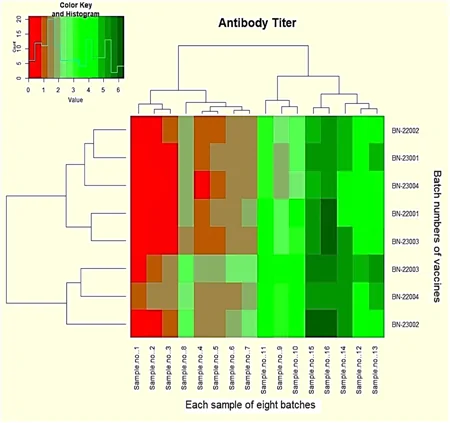

## 1 INTRODUCTION

The neurotropic rabies virus (RABV), also known as rabies lyssavirus, is the cause of rabies in both people and animals. Rabies can be spread by contact with animal saliva and, less frequently, human saliva. Many mammalian species have been shown to be infected by it in the wild, and in the lab, it has been discovered that cell cultures from mammals, birds, reptiles, and insects can also be infected.^1^ On all continents, with the exception of Antarctica, rabies has been reported in more than 150 countries.^2^ The majority of sickness cases are documented in Asia and Africa; however, in the past 10 years, some instances have also been reported in Europe, particularly among returning travelers.^3^

RABV contain an envelope and a single‐stranded, negative‐sense RNA genome. The virus's RNA genome has five genes in a highly conserved arrangement. These genes produce the viral RNA polymerase (L), the nucleoprotein (N), the phosphoprotein (P), the matrix protein (M), and the glycoprotein (G).^4^ The length of the entire genome sequence varies from 11,615 to 11,966 nt.^5^

Every year, rabies kills tens of thousands of people, mostly in Asia and Africa, and 40% of those killed are children under the age of 15. Dogs are the primary cause of human rabies mortality, accounting for up to 99% of all human cases of rabies. Postexposure prophylaxis (PEP), a series of rabies vaccinations, and, if necessary, the delivery of rabies immunoglobulin or monoclonal antibodies are all options for persons who may have been exposed to a potentially rabid animal. Globally, rabies is thought to cost $8.6 billion annually.^6^

On July 6, 1885, Louis Pasteur administered the first dose of his rabies vaccine to a patient who had been bitten by a human.^7^ In 1967, work on the human diploid cell rabies vaccine (HDCV) began. The attenuated Pitman‐Moore L503 strain of the RABV is employed to create the inactivated HDCV.^8^

Along with these advancements, purified chicken embryonic cell vaccines (CCEEV) and purified, Vero cell rabies vaccinations are currently offered by the WHO.^9^ As advised by the manufacturer's, PEP and PrEP (preexposure prophylaxis) regimens necessitate a series of vaccination doses. As of right now, the majority of vaccine makers advise (i) a three‐dose 1‐site IM regimen for PrEP and (ii) for PEP a 1‐site IM five‐dose regimen on Days 0, 3, 7, 14, and 28 or four‐dose Zagreb regimen (2‐site IM on Day 0 and 1‐site IM on Days 7 and 21).^10^ In addition, some manufacturer's provide the 2‐site ID Thai Red Cross regimen with four clinic visits on Days 0, 3, 7, and 28 for PEP.^6^, ^11^ CCEEVs are safe for use in both humans and animals and use inactivated RABV that has either been generated from embryonated eggs or in cell cultures.^9^, ^11^ The first WHO‐approved purified verocell rabies vaccines, such as VERORAB and SPEEDA.^12^, ^13^ There are now two kinds of oral rabies vaccination for wildlife in use: modified live virus SAG2 and SAD B19.^14^ Second, the recombinant vaccinia virus encoding the gene for the rabies glycoprotein, it is primarily employed in the USA and Western Europe.^15^ Repeated vaccinations can maintain a high degree of cell‐mediated cytotoxic activity, and the presence of antibodies has no impact on the secondary activation of sensitized lymphocytes.^16^

The agar gel precipitation test, known as single radial immunodiffusion (SRID), where agar gel is filled with an antibody with known specificity, and a sample containing the target antigen is deposited in one of the gel's wells.^17^ The original rapid fluorescent focus inhibition test (RFFIT) has been modified into a micro‐test known as the FAVN test (fluorescent antibody virus neutralization test). The live viral FAVN test is used to assess if an animal has enough rabies antibodies after immunization. Antibody concentrations above 0.5 IU/mL are considered sufficient for rabies defense.^18^ The National Institutes of Health (NIH) established the NIH test, is a in vivo test which is used to evaluate the potency of the rabies vaccine. In this experiment, mice are first inoculated with rabies vaccine before being exposed to the RABV, to assess the level of protection provided by inactivated rabies vaccinations.^19^

The study aimed to evaluate the quality and efficacy of Bangladeshi‐manufactured rabies vaccines comparing with an internationally renowned brand. Are locally manufactured vaccines efficacious and reliable? We performed WHO‐approved in vivo and in vitro methods to determine the quality through potency, immunogenicity, and efficacy studies.

## 2 MATERIALS AND METHODS

### 2.1 Study design

Samples were provided from manufacturer to National Regulatory Authority for lot release purpose. Then samples were analyzed at The National Control Laboratory, Directorate General of Drug Administration, under the Ministry of Health and Family Welfare, Bangladesh. From March 2020 through May 2023, a total of three laboratories, one animal house, and five veterinary clinics were involved. Rabies vaccine is a freeze‐dried form of the inactivated RABV produced on Vero cells. The vaccine is a clear, colorless, sterile solution after reconstitution with water for injection (WFI). The study was designed to analyze 20 separate batches of rabies vaccine. Three immunochemical methods were employed. In every method there were two groups: the test group and control group. Sixteen articles were randomly chosen for each test group. For positive control group, we used reference vaccine; WFI was used for negative control group. Dogs was involved in FAVN and mice were involved in NIH in vivo test method for potency. Sixteen separate animals for test group, one for positive control and one for negative control, were designed as experimental single unit. A total of 360 dogs, 18 for each batch (16 for test group and 2 for control group), were used in FAVN test.^20^, ^21^ The dogs were not less than 9 months of age and not below 10 kg. They were not vaccinated before, and blood was collected within 120–180 days of vaccination. Both dogs and bitches were selected. A total of 2920 mice, 146 for each batch (96 for immunization, 48 for back titration, and 2 for control group) were used in NIH test).^22^, ^23^ Healthy Swiss albino female mice of 11–15 g weight and 21–27 days of age were selected for NIH test. Dogs and mice were selected randomly after quarantine and initial health checkup. Only analysts and medical technologists were aware of the control group and test group. Animal caretakers and other lab personnel were not informed about it. After NIH challenge test, we observed typical clinical signs like ruffled fur, hunched back, slow or circular movement, loss of alertness, shaky movements, trembling, convulsions, paralysis, moribund state, and death. Statistical analysis was performed by Combistat software (version 6.01). Experimental procedures are described in material and method section. The results of our experiment are described in Tables 1, 2−3.

**Table 1:** Results of the SRID tests of samples attained from 20 batches of rabies human vaccines.

Batch no	No of article	Potency of standard (mean ± SD) IU/mL	Potency of sample (mean ± SD) IU/mL	Coefficient of variation (%) of standard	Coefficient of variation (%) of sample	Variance of standard	Variance of sample	Pearson's correlation coefficient	Regression (*R* ^2^)	Acceptance value of potency	*p* Value (two‐tailed *t*‐test)	Satisfactory percentage (%)
BN‐20001	16	5.355 ± 0.451	5.126 ± 0.759	8.422035481	14.806867							100
BN‐20002	16	5.465 ± 0.451	5.305 ± 0.713	8.252516011	13.440151							100
BN‐21001	16	5.355 ± 0.451	5.282 ± 0.682	8.422035481	12.911776							100
BN‐21002	16	5.41 ± 0.45	5.314 ± 0.632	8.31792976	11.893113							100
BN‐21003	16	5.41 ± 0.45	5.219 ± 0.640	8.31792976	12.262886							100
BN‐21004	16	5.355 ± 0.451	5.211 ± 0.684	8.422035481	13.126079							100
BN‐21005	16	5.41 ± 0.45	5.274 ± 0.681	8.31792976	12.9124							100
BN‐21006	16	5.355 ± 0.451	5.22 ± 0.689	8.422035481	13.199234							100
BN‐21007	16	5.465 ± 0.451	5.346 ± 0.608	8.252516011	11.372989							100
BN‐22001	16	5.355 ± 0.451	5.22 ± 0.543	8.422035481	10.402299	0.003144	0.00566	0.7	0.4641	2.5 IU/mL	.00000000001268	100
BN‐22002	16	5.355 ± 0.451	5.16 ± 0.657	8.422035481	12.732558							100
BN‐22003	16	5.355 ± 0.451	5.094 ± 0.644	8.422035481	12.642324							100
BN‐22004	16	5.355 ± 0.451	5.133 ± 0.662	8.422035481	12.896941							100
BN‐22005	16	5.41 ± 0.454	5.291 ± 0.637	8.391866913	12.037037							100
BN‐22006	16	5.41 ± 0.454	5.256 ± 0.574	8.391866913	10.920852							100
BN‐22007	16	5.355 ± 0.451	5.155 ± 0.640	8.422035481	12.415131							100
BN‐22008	16	5.465 ± 0.451	5.221 ± 0.542	8.252516011	10.381153							100
BN‐23001	16	5.52 ± 0.44	5.268 ± 0.493	7.971014493	9.3583903							100
BN‐23002	16	5.3 ± 0.44	5.085 ± 0.576	8.301886792	11.327434							100
BN‐23003	16	5.465 ± 0.451	5.261 ± 0.613	8.252516011	11.651777							100

Abbreviation: SRID, single radial immunodiffusion.

**Table 2:** The sensitivity of the FAVN method of assaying sera from dogs immunized with rabies vaccine and the quantity of antibody titers presented with statistical analysis.

Batch no	No of vaccinated dogs	Day of antibody test after first vaccination	Titer IU/mL average ± SD					Titer IU/mL average ± SD	Satisfactory percentage (≥0.5 IU/mL)	CV	Variance
			<0.5 IU/mL	0.5–1 IU/mL	1–2 IU/mL	2–5 IU/mL	≥5 IU/mL				
BN‐20001	16	120−150	1 (6.25%)	2 (12.5%)	5 (31.25%)	6 (37.5%)	2 (12.5%)	2.7325 ± 1.77	93.75	0.647914203	0.063155058
BN‐20002	16	120−180	0 (0%)	2 (12.5%)	6 (37.5%)	6 (37.5%)	2 (12.5%)	2.591875 ± 1.63	100	0.626446737	0.064823885
BN‐21001	16	120−150	0 (0%)	1 (6.25%)	4 (25.0%)	7 (43.75%)	4 (25.0%)	3.281875 ± 1.74	100	0.530462762	0.062323592
BN‐21002	16	120−180	0 (0%)	0 (0%)	6 (37.5%)	7 (43.75%)	3 (18.75%)	3.036875 ± 1.60	100	0.526802108	0.058381107
BN‐21003	16	120−180	1 (6.25%)	2 (12.5%)	6 (37.5%)	5 (31.25%)	2 (12.5%)	2.56625 ± 1.73	93.75	0.676610626	0.060991107
BN‐21004	16	120−180	1 (6.25%)	1 (6.25%)	4 (25.0%)	7 (43.75%)	3 (18.75%)	3.060625 ± 1.87	93.75	0.614054853	0.056945927
BN‐21005	16	120−150	1 (6.25%)	2 (12.5%)	5 (31.25%)	5 (31.25%)	3 (18.75%)	2.703125 ± 1.90	93.75	0.706145549	0.059636745
BN‐21006	16	120−180	2 (12.5%)	2 (12.5%)	4 (25.0%)	6 (37.5%)	2 (12.5%)	2.389375 ± 1.70	87.50	0.712767839	0.060437713
BN‐21007	16	120−150	0 (0%)	1 (6.25%)	3 (18.75%)	9 (56.25%)	3 (18.75%)	2.66875 ± 1.61	100	0.60382208	0.038758689
BN‐22001	16	120−180	1 (6.25%)	1 (6.25%)	5 (31.25%)	7 (43.75%)	2 (12.5%)	2.71 ± 1.68	93.75	0.622677372	0.03275483
BN‐22002	16	120−150	0 (0%)	2 (12.5%)	5 (31.25%)	8 (50.5%)	1 (6.25%)	3.09125 ± 1.71	100	0.553639936	0.026335488
BN‐22003	16	120−180	2 (12.5%)	1 (6.25%)	4 (25.0%)	6 (37.5%)	3 (18.75%)	2.961875 ± 1.65	87.50	0.558414424	0.028892429
BN‐22004	16	120−180	0 (0%)	1 (6.25%)	5 (31.25%)	9 (56.25%)	1 (6.25%)	2.960625 ± 1.41	100	0.478174442	0.031841992
BN‐22005	16	120−180	0 (0%)	2 (12.5%)	6 (37.5%)	7 (43.75%)	1 (6.25%)	2.93875 ± 1.74	100	0.594718666	0.035386368
BN‐22006	16	120−150	1 (6.25%)	1 (6.25%)	4 (25.0%)	8 (50.0%)	2 (12.5%)	2.9775 ± 1.728	93.75	0.580541401	0.03886594
BN‐22007	16	120−180	1 (6.25%)	2 (12.5%)	5 (31.25%)	5 (31.25%)	3 (18.75%)	2.8125 ± 1.36	93.75	0.48241696	0.044636188
BN‐22008	16	120−150	2 (12.5%)	1 (6.25%)	4 (25.0%)	4 (25.0%)	5 (31.25%)	2.9425 ± 1.60	87.50	0.544904619	0.032226831
BN‐23001	16	120−180	1 (6.25%)	2 (12.5%)	5 (31.25%)	6 (37.5%)	2 (12.5%)	3.025 ± 1.54	93.75	0.511167458	0.022751823
BN‐23002	16	120−150	0 (0%)	2 (12.5%)	5 (31.25%)	8 (50.0%)	1 (6.25%)	3.3875 ± 1.59	100	0.470798888	0.003562598
BN‐23003	16	120−180	1 (6.25%)	1 (6.25%)	6 (37.5%)	7 (43.75%)	1 (6.25%)	3.268125 ± 1.59	93.75	0.487861107	0

Abbreviation: FAVN, fluorescent antibody virus neutralization.

**Table 3:** NIH test results of tested batches of rabies human vaccine after 14 days of inoculation.

Serial no	Batch no	No of challenged mice	Potency of standard IU/SHD	Mean of standard potency	Potency of sample IU/SHD	Mean of sample potency	Minimum potency set by WHO	Regression *R* ^2^	*p* Value	CV of sample	CV of standard	Satisfactory percentage (%)
1	BN‐20001	16	6.22	5.899 ± 0.52 IU/SHD	4.78	4.777 ± 1.13 IU/SHD	2.5 IU/SHD	0.972	.00026	0.2364	0.0842	100
2	BN‐20002	16	6.48		5.51					0.2051	0.0809	100
3	BN‐21001	16	6.08		4.24					0.2665	0.0862	100
4	BN‐21002	16	5.42		3.66					0.3087	0.0967	100
5	BN‐21003	16	5.96		5.18					0.2181	0.0879	100
6	BN‐21004	16	6.14		4.38					0.2580	0.0853	100
7	BN‐21005	16	5.34		2.86					0.3951	0.0981	100
8	BN‐21006	16	6.52		6.18					0.1828	0.0804	100
9	BN‐21007	16	5.56		3.74					0.3021	0.0942	100
10	BN‐22001	16	6.84		6.18					0.1828	0.0766	100
11	BN‐22002	16	5.27		5.02					0.2251	0.0994	100
12	BN‐22003	16	5.42		4.36					0.2592	0.0967	100
13	BN‐22004	16	6.28		6.03					0.1874	0.0834	100
14	BN‐22005	16	5.47		4.94					0.2287	0.0958	100
15	BN‐22006	16	5.13		2.86					0.3951	0.1021	100
16	BN‐22007	16	6.16		5.73					0.1972	0.0851	100
17	BN‐22008	16	5.92		4.85					0.2330	0.0885	100
18	BN‐23001	16	6.68		6.37					0.1774	0.0784	100
19	BN‐23002	16	5.17		2.95					0.3831	0.1014	100
20	BN‐23003	16	5.92		5.72					0.1976	0.0885	100

Abbreviation: NIH, National Institutes of Health.

### 2.2 Estimation of glycoprotein content in rabies vaccine by SRID method

Briefly, Tris buffer of 0.1 M (MERCK; Catalogue Number, 648315); sodium azide (Sigma‐Aldrich; CAS 26628‐22‐8); sodium chloride (Scharlau Lab; EC number: 231‐598‐3); staining solution, Coomassie Brilliant Blue (Thermo Scientific and Thermo Fisher; Catalog number: 20279); ethanol (DUKSAN Pure Chemicals Co., Ltd.; CAS No. 64‐17‐5); acetic acid (DAEJUNG Chemicals; CAS No.: [64–19–7]); glycerol (Merck KGaA; CAS no. 104091) was prepared for SRID as per Mayner RE, Williams MS,^24^, ^25^ and specific manufacturer's guideline. After reconstituting, the standard was serially diluted to 1:4, 1:8, 1:16, 1:32, and 1:64 of glycoprotein in 0.9% NaCl. Sample was also reconstituted with 1.0 mL WFI and diluted as like standard. One percent agarose solution was prepared and well of 3 mm diameter was created for every dilution. Four microliters (max) of the reference standard and sample was added to the wells. Ten minutes later, a foil‐wrapped tray with clotted gel was transferred to the moisture chamber. After 24 h of incubation, the clotted gel was separated and transferred to a staining tray. After staining with staining solution, the clotted gel was taken out and washed with destaining solution for 15 min. After drying, the diameter of the diffusion circle of the standard and sample was measured by an automatic zone reader. The zone area was calculated with calibrated Microsoft Excel according to formula (formula is in data not for publication).

### 2.3 Serum antibody test: The FAVN test

Virus neutralization test can be performed in cell culture. The antibody titers of serum samples were measured by the FAVN test performed according to the method described by Cliquet et al.^20^ The challenge virus suspension (CVS)‐11 (CVS‐11, ATCC VR‐959) virus was produced in BHK‐21 C13 cells. After culturing BHK‐21 C13 cells in DMEM (Thermo Fisher Scientific; Cat No. 11965092) media, cells were then infected, grown, and harvested within uninfected golden hamster (BHK‐21 C13, ATCC No. CCL‐10™) cells. The TCID_50_ test was employed to calculate the viral titer. In brief, threefold serial dilutions of both the test sera and the control sera were prepared in microplates. Each serum dilution was tested in quadruplicate, with one control plate and four plates with sera to be tested. 50 μL (100 TCID_50_) of challenge RABV CVS‐11 stored at −80°C was also added to each well. One dilution from this tube is performed using DMEM supplemented with 10% fetal calf serum (Sigma‐Aldrich; Chemie GmbH Product No. A4781). The microplates are incubated for 1 h at 37°C in a 5% CO_2_ humidified incubator. Cells are maintained in GMEM supplemented with 10% FCS and antibiotics. The concentration of cells was counted using a haemocytomer (Neubauer chamber). The final concentration was maintained to get 4 × 10^5^ cells/mL. Then 50 μL of BHK‐21 cell suspension was added to each well, and the plates were incubated for 48 h at 37°C. After fixing the cells in 80% cold acetone for 30 min at room temperature, the plates were stained by adding fluorescein isothiocyanate (FITC) (Sigma‐Aldrich; cat no. 34321)‐conjugated antirabies monoclonal antibody, incubating at 37°C for 30 min, and then washing with phosphate buffer saline (PBS)‐Tween 20. The plates were examined through fluorescence microscopy (ZEISS Axioscope 5; Carl Zeiss). The reading is qualitative; presence or absence of fluorescent foci in the cells was recorded. Fifty percent endpoints were calculated as the inverse of the highest dilution with 2/4 cells showing no fluorescence, using the Spearman's−Kärber formula (formula is in data not for publication). Antirabies serum (AFSSA) was included as a positive control and blood from non‐vaccinated dog was considered as negative control in all assays, and data were calculated as international units (IU)/mL of RABV‐neutralizing antibody.^21^, ^26^

### 2.4 NIH test (mice challenge test)

The mouse challenge test was performed in accordance with The British Pharmacopeia and WHO.^22^, ^23^

#### 2.4.1 Reagent preparation

NaCl (Scharlau Lab; product code: SO0225), KCL (MERCK KGaA; Cat no.: 1049360500), KH_2_PO_4_ (MERCK KGaA; Cat no.: 1048731000), and Na_2_HPO_4_. 2H_2_O (Scharlau Lab; product code: SO0339) was taken in a 1000 mL volumetric flask and volume filled to the marked level by purified water to prepare phosphate buffer. PBS for vaccine was prepared by dissolving NaCl, Na_2_HPO_4_ (Scharlau Lab; product code: SO0227), and KH_2_PO_4._ Solution pH was adjusted to 7.2−8.0 and autoclaved. PBS containing 2% fetal bovine serum (FBS) was formulated using FBS (Sigma‐Aldrich; ES‐009‐B or Thermo Fisher Gibco FBS‐16000044) and mixed with PBS. The volume was filled up to the mark with PBS for viruses. Rabies CVS was prepared with an ampoule of instant thawed frozen virus (ATCC VR 959; CVS‐11) and diluted with 2% horse serum diluent.

#### 2.4.2 RABV titer detection

The median effective dose (ED 50) was determined with 6‐week‐old mice. A 10‐fold serial dilution of CVS supernatant was prepared, and 0.03 mL of each CVS was intracerebrally inoculated in groups of 10 mice. Mice were observed for any deaths in the first 5 days between the 1st and 14th days. On the 14th day, antibody titers were tested using the Reed‐Muench formula (test and data not for publication).

For the first immunization, test and standard vaccines were reconstituted with 1 mL WFI. Then, using PBS, three fivefold dilutions (1:25, 1:125, and 1:625) of the standard and test vaccines were prepared.

#### 2.4.3 Immunization

After the completion of the quarantine period first immunization was done, 16 healthy Swiss Albino Mice (8 male and 8 female) were selected for each dilution. Each dilution was administered intraperitoneally to each mouse in 1.0 mL. Every mouse was kept in a separate case with proper labeling. On the 7th day after the first immunization we did second immunization, the second immunization was performed in the same manner.

#### 2.4.4 Challenge test

On the 14th day after the first immunization, CSV suspension was diluted to get the challenge virus with a titer of 50 LD_50_/0.03 mL. Then, on 14th day of first immunization, immunized mice and a virus suspension containing about 50 LD_50_/0.03 mL titer were taken. 0.03 mL of virus suspension was injected into each mouse. Mice were kept in an observation room. Challenge virus with a titer of 50 LD_50_/0.03 mL is considered 10°. 0.2 mL of 10° CVS suspension was mixed with 1.8 mL of PBS in a 15 mL falcon tube to make a 10^−1^ dilution. Then 10^−2^, 10^−3^, and 10^−4^ dilutions of the challenging virus for virulence titration (back titration) were performed. On the same day, healthy mice weighing 11−15 g were taken. Then 0.03 mL of challenge virus from each dilution (10^−1^, 10^−2^, 10^−3^, and 10^−4^) was injected intracerebrally into each mouse.

#### 2.4.5 Observation

Mice were observed daily for 14 days starting from the date of challenge, and deaths were recorded. The mice that died or manifested typical signs of encephalopathy on or after the 5th day following the challenge were included.

#### 2.4.6 Reference vaccine

The Pitman Moore strain of RABV, generated in the Nil‐2 cell line and inactivated using ß‐propiolactone, served as the basis for the freeze‐dried vaccine known as Biological Reference Preparation (BRP) batch No. 5, which is now being distributed by the European Directorate for the Quality of Medicines. The assigned titer for this reference vaccine is 10 IU per vial.

#### 2.4.7 Statistical analysis and validation of test results

The all‐or‐none response is the foundation of the NIH potency test. As a result, a parallel‐line model with at least three points for BRP No. 5 and the vaccination under consideration was employed. Statistical analysis was conducted with CombiStat software version 6.01.

## 3 RESULT

**Figure 1:**
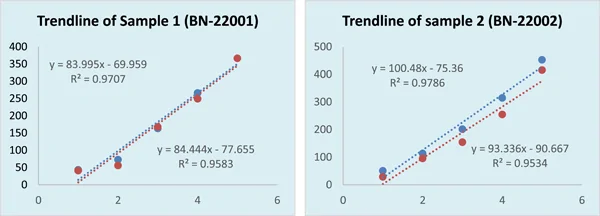
Straight line to show trend of concentration (*x*‐axis) versus zone area (*y*‐axis). Straight line equation denotes slope of the line and correlation coefficient of tested sample. Here, blue color denotes the standard and red color denotes the sample.

**Figure 2:**
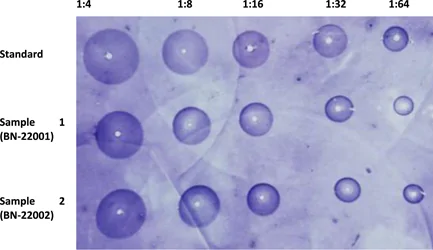
Single radial immunodiffusion assay shows zone produced by rabies virus glycoprotein. Diameter of the precipitation zone for different dilution is shown here.

Table 1 shows the potency of the vaccine as determined by SRID. The average potency of 16 tested articles is expressed with a standard deviation. This is an intra‐batch comparison for a uniform, repeatable result. The potency of the standard is either 4.97 or 5.85 because it is a known concentration. Although the acceptance potency is 2.5 IU/mL, we always found a potency value of ≥4.0 IU/mL, which is a great unboundedness. The consistency of the standard was also checked for uniformity and repeatability. The mean of standard and sample was never below 2.5 IU/mL. Even the minimum value of each batch was greater than the acceptance value. Inter‐batch results were compared with several statistical analyses. Variance was analyzed to find the degree of dispersion of the mean of the standard and samples. The precipitation circle area was measured in millimeters. The variance on average potency of 20 batches did not show any significant difference. To determine the linear relationship between standard and sample, we used the Pearson's correlation coefficient. We found a correlation coefficient value of 0.7, which means there is a significant and positive correlation between the potency of the standard and the potency of the sample. When we checked the relative dispersion of 20 batches around the mean potency through the coefficient of variation (CV), we found the variation coefficient to be less than 15% every time. In Table 1, we excerpt linear regression (*R* ^2^) between sample and standard. The regression value was calculated at 0.4641, with a well‐accepted significance value of 0.001319449. The probability value of a two‐tailed *t*‐test is less than 0.001, which is statistically highly significant. In Figure 1, we revealed two trend lines as a vicarious expression of linear regression. The regression of every single test sample, intra and inter‐batch is quite different. So, we took the mean of 16 samples from every batch. Figure 2 is a captured photo from the zone reader. The diameter of the precipitation zone was gradually decreased according to dilution.

### 3.1 Potency of sample determined by SRID method

**Figure 3:**
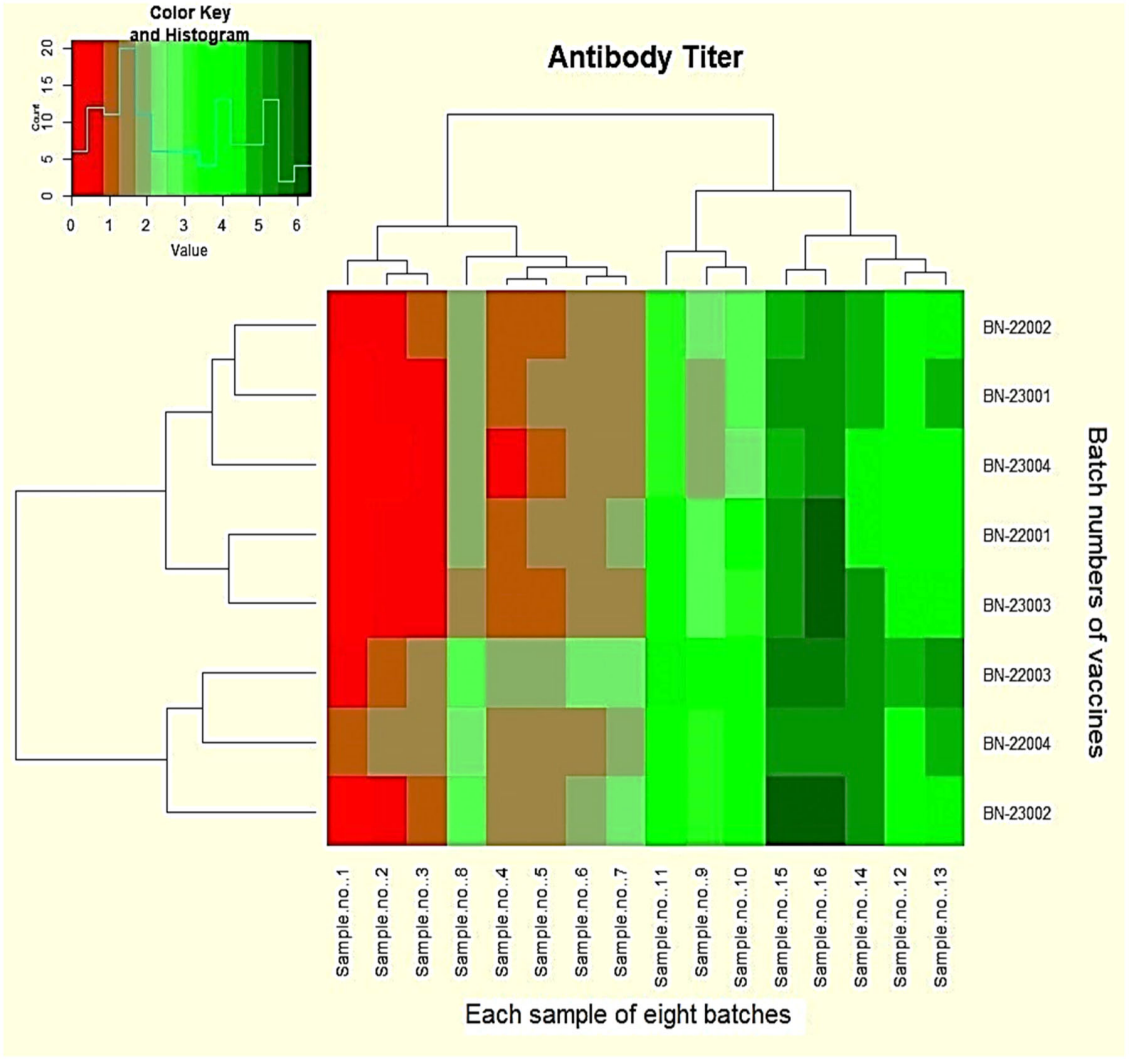
Heatmap represents the antibody titer quantity. Red blocks reveal the antibody titer ≤0.5 IU/mL. Dark red blocks indicate the value of ≥0.5. All other green blocks have a value of ≥1.0 to 6.5.

**Figure 4:**
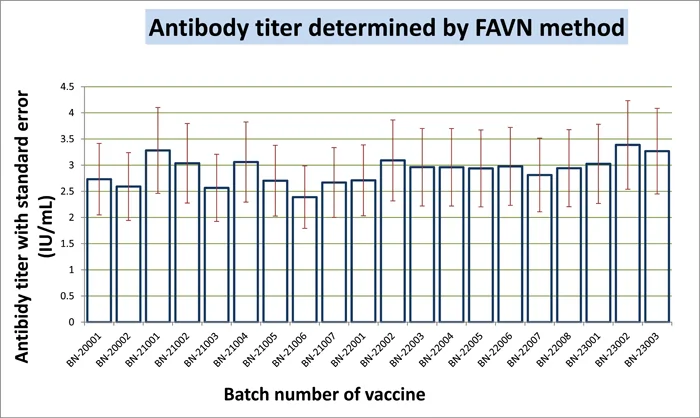
Detection of rabies antibody by the FAVN test method, results in a bar graph with a standard error. FAVN, fluorescent antibody virus neutralization.

FAVN is also an in vitro method that helps determine immunogenicity through antibody titer quantification. We categorized our quantification into five categories: <0.5, 0.5−1, 1−2, 2−5, and ≥5 IU/mL (Table 2). Twenty batches were tested; each batch contained 16 articles to inject into 16 dogs that had not been immunized before. After vaccination, the first blood samples were collected within 120−180 days. The highest number of dogs generated antibodies within 2−5 IU/mL, followed by 1−2 IU/mL. The threshold limit for antibody titer for the FAVN method is ≥0.5 IU/mL according to the WHO guideline. But out of 20 batches, 12 batches exhibit one or two samples with a potency less than 0.5 IU/mL, which is 6.25%−12.5% of the 16 articles in each batch. Thus, a 100% satisfactory result could not be achieved for all 20 batches, although the unsatisfactory percentage is very minimal. We found a 100% intra‐batch satisfactory percentage for eight batches. Meanwhile, other batches had a satisfactory range of 87.5−93.75. If the number of articles could be increased, the result would be more statistically balanced. If we consider the mean potency of 16 articles, it is more acceptable than the potency of an individual article. All mean values are greater than 2.0 IU/mL, which is above threshold value. The inter‐batch mean value was calculated within 2.39−3.38 IU/mL. The standard deviation of antibody titer shown with the mean in Table 2 indicates that the data is spread out from the mean with a great deal of variation. The variance is between 0 and 0.065, with a median value of 0.0388. We calculated the CV to find the dispersion of a probability distribution. So, our minimum and maximum correlations of variation were, respectively, 0.471 and 0.713, with a median of 0.569. Here, CV indicates that the existing variability is high and values are dispersed within a greater range. In Figure 3, we show the mean value of the antibody titer with a standard error. The figure graphically expressed the mean value of every batch. But we cannot deduce any unsatisfactory result from this graphical presentation. The statistical analysis for uniformity, linearity, and reproducibility showed significant differences within the same batches, but the mean of inter‐batch data showed statistically significant data (*p* > .05). This intra‐batch result is discordant for a greater range, standard deviation, and variance. In Figure 4, we presented microscopy images of high and low antibody titers with BHK‐21 cells and CSV‐11 viruses. Negative control of BHK‐21 cells were fixed with acetone and stained with FITC without the CSV‐11 virus is also shown in Figure 4. The transfected BHK‐21 cells were visualized after 48 h of incubation with the CSV‐11 virus under fluorescence microscopy to observe green fluorescence. In Figure 4A, green fluorescence illuminates against a dark red background. The result is qualitative, which means only presence or absence can be detected; quantification cannot be measured by microscopy. But a low antibody titer gives less green fluorescence (Figure 4A), while a higher antibody titer provides a green background (Figure 4B). When BHK‐21 cells were fixed with acetone and stained with FITC without CVS‐11 virus infection, no antibody was produced, and green fluorescence was emitted against a dark red background (Figure 4C). We also performed a negative control test (data not shown) in every batch. Blood from a non‐vaccinated dog was infected with CSV‐11, like other samples and positive controls. Negative control produced a 0.03−0.18 IU/mL antibody titer against the virus in 20 tests. In some cases, antibody titers checked at Days 120−180 had a higher mean titer than those checked at Days 120−150, but this concordance was not always certain. In a few cases, 120−150 days of incubation had a greater antibody titer mean than 120−180 days. So, days of postvaccination might not always be a determinant for producing a quantity of antibody titer, while other variables like age, race, size, and breeding have a probable influence. When we calculated antigen (glycoprotein) potency by the SRID method, we found a uniform and significant amount of potency (≥2.5 IU/mL), but we could not generate antibodies with a uniform quantity.

**Figure 5:**
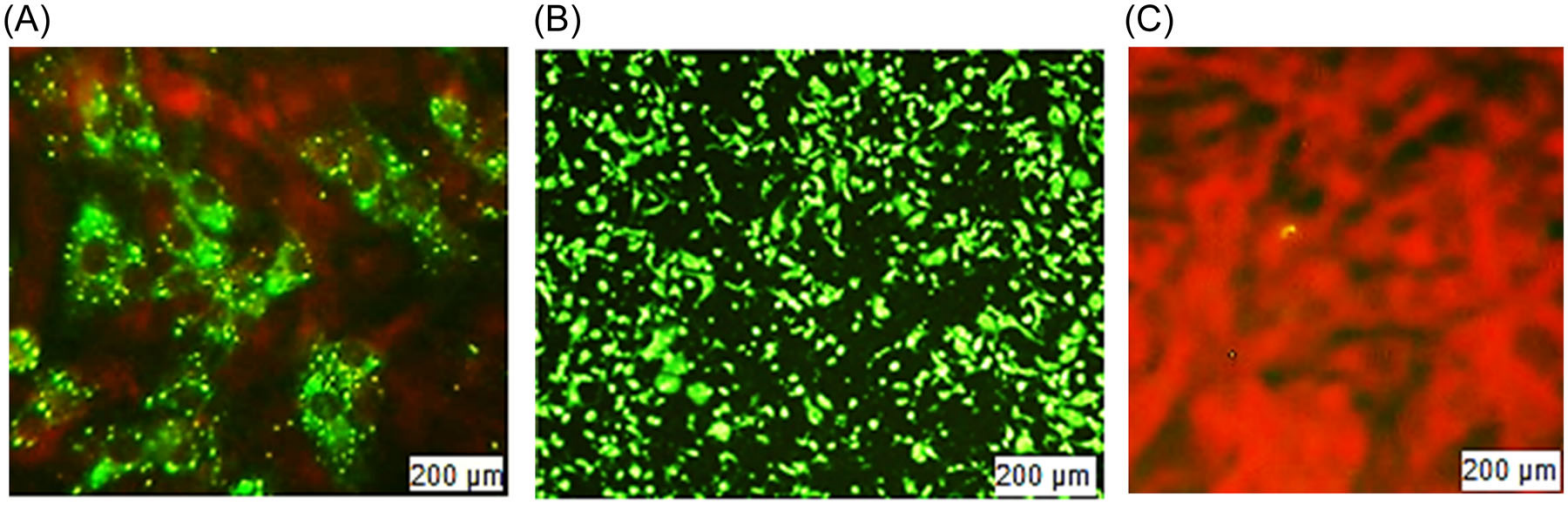
Picture from ZEISS Axioscope 5 fluorescent microscopy, captured by Axiocam 208 and processed by ZEN imaging software after 48 h of incubation. Fluorescent antibody virus neutralization (FAVN) test. (A) (Higher dilution and low titer) BHK‐21 cells stained with FITC, after 48 h of transfection with CSV‐11. (B) (Lower dilution and higher titer) after 48 h of transfection with CSV‐11. (C) Negative control, BHK‐21 cells were fixed and stained with acetone and FITC but CVS was not added. CVS, challenge virus suspension; FITC, fluorescein isothiocyanate.

**Figure 6:**
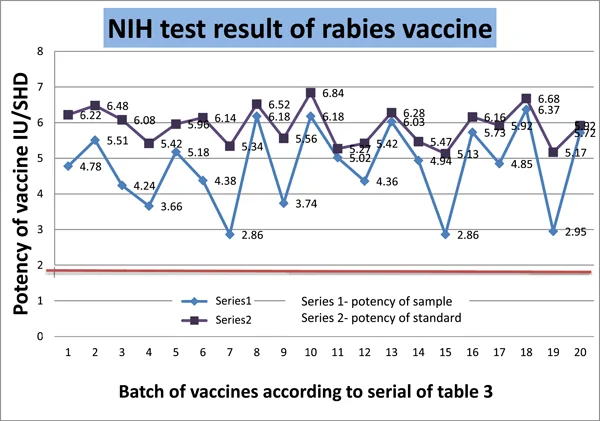
Potency of rabies vaccine determined by NIH method presented in chart‐line diagram. NIH, National Institutes of Health.

The NIH assay is presently a crucial requirement for vaccine distribution and is used to evaluate the effectiveness of rabies vaccination. This test is totally in vivo because it entails immunizing mice and then exposing them to a viral challenge. The test design comprised of 20 batches; each batch contained 16 articles to inject into 16 mice. The potency of the standard was very consistent, having a mean of 5.899 IU/SHD with a standard deviation of 0.52 IU/SHD (Table 3). The minimum potency of human rabies vaccine is 2.5 IU/mL. Every sample had a potency greater than 2.5 IU/SHD. The mean potency value of 20 batches is 4.78 IU/SHD, with a standard deviation of 1.13 IU/SHD. None of the 20 batches exerted any value ≤2.5 IU/SHD which indicates no unsatisfactory result. Minimum potency we got 2.86 IU/SHD, and maximum potency was 6.37 IU/SHD. Although the minimum value was close to the threshold value, the mean and median values were, respectively, 4.77 IU/SHD and 4.895 IU/SHD, which implies a good central tendency of our acquired data. Albeit, mean potency of the standard is higher than the sample potency, the difference is comparable. We calculated the *p*‐value against a two‐tailed *t‐*test, assuming two samples have equal variances. Our calculated *p*‐value was .00026, which is statistically very significant. Besides the standard deviation, we calculated the CV to measure the dispersion of data points. The CV of the sample was always greater than that of the standard. It means the data of the sample is more spread out compared with the more uniform data of the standard. If we look at the potency of our 20 samples, the CV is between 17.74% and 39.51%, which imparts a higher variance and standard deviation. We analyzed regression to understand the relationship between the potency of the standard and the potency of samples. The regression value (*R* ^2^) was 0.972, which is greater than 0.95 and statistically reliable. So, the in vivo test method confirms that the vaccine is able to provide complete protection against the RABV. All 20 batches were complaint in NIH potency test according to survival capability with different dilutions of vaccines. Inter‐batch potency comparison through uniformity, linearity, and repeatability showed that our data was uniform, regression was linear, and the mean with standard deviation was concordant. In Figure 5, a trend chart of the potency of the standard and sample is visualized. All samples and standards passed, which is above the threshold limit (Figure 6).

## 4 DISCUSSION

In recent years, rabies cases in humans have risen steadily in developing nations. In a national epidemiological investigation, just 2% of patients had been infected by other animals, while 95% of patients had been bitten by dogs.^27^ To eliminate human rabies in China and other emerging nations, it is crucial to manage and prevent canine and human rabies. The only way to prevent, manage, and eliminate rabies in wild animals in areas with substantial wildlife transmission is through mass vaccination campaigns.

In our SRID result shown in Table 1, it is clear that the potency of the standard and the potency of the sample have a linear relationship. The results of 20 batches of SRID were 100% satisfactory compared with the WHO threshold limit. Moreover, the mean value of 16 samples from every batch reveals greater potency than stipulated.^28^ So it is easy to perceive that SRID approaches could potentially be useful for assessing rabies vaccine both quantitatively and qualitatively, as well as for assaying the rabies glycoprotein antigen for research purposes. SRID tests are quick and easy to perform, and they have the benefit of offering an alternate assay system, which lessens the need for animal protection tests using infectious viruses.^29^ SRID assays might be useful for determining the antigen concentration of vaccines in vitro and could supplement data from in vivo tests, which are prone to large variations. The coefficients of variation for the mean of the potency from all 20 batches were between 10% to 14%, indicating good agreement between the potency measured by the SRID method. This SRID method is less cumbersome and less time‐consuming, but its reproducibility and linearity are remarkable. Ferguson et al.^30^ described the prime limitation of SRID, this method is not capable of determining the immunogenicity of a host. Williams et al.^31^ also found a stable, sensible, and reproducible result using variance and regression analysis while determining the potency of a viral vaccine. Volokhov et al.^32^ found the enzyme‐linked immunosorbent assay (ELISA) technique to have more throughput, seem quicker and more dependable, and might be a simpler alternative. Rajasekaran^33^ observed that 42.31% (132/312) of the 312 wild boar samples that underwent ELISA testing for rabies showed antibodies. But only 28.4% (98/345) of the fox samples showed rabies antibodies by ELISA. Before the final lot of the vaccine is tested, the glycoprotein content during manufacturing is a helpful predictor of its potency, which can be tested using the SRID test; however, ELISA approaches, such as indirect and immuno‐capture kinds, are showing high throughput, seem faster and more reliable, and may be simpler alternatives.^34^, ^35^ Compared with Ferguson et al. and Williams et al., the potency of our result has a higher mean and a lower SD value but a greater CV%, regression, and correlation coefficient. Overall satisfactory percentage is also higher for our glycoprotein content.

There are some widely accepted and WHO‐approved methods for the quantification of rabies neutralizing antibody titers: the RFFIT, the mouse neutralization test, the ELISA, and the FAVN test.^21^, ^23^, ^36^ The FAVN test is a modified technique, an adjusted RFFIT, that is one of the accepted WHO procedures for figuring out antibody titers. The number of fluorescent foci in virus‐infected cells must be manually counted with RFFIT,^37^ but FAVN is unbiased, enables automated high‐volume detection. Despite the fact that the ERA, Hep‐Flury, and CTN‐181 are safer than CVS‐11, it is preferable to assess the efficacy of vaccinations using a pathogenic challenge virus, such as the CVS‐11 strain, rather than the vaccine strain itself. In RABV neutralizing antibody tests, the CVS‐11 strain has received widespread approval as a challenge virus.^38^ We used the CVS‐11 virus strain and easily observed it in infected cells with fluorescence microscopy. By the FAVN test, we detected virus‐neutralized antibodies at a great level in vaccinated dog's blood. The result is satisfactory according to the mean value. Moreover, our inter‐batch potency result exhibited excellent agreement with WOAH and WHO references. The mean value of each batch is way greater than the threshold limit. Although we did not test this vaccine against humans, Xue et al.^39^ found that vaccine from the same batch, in comparison to human serum samples, canine serum samples had a superior agreement with detection. Because the titers obtained are larger than those from standard FAVN, it is important to carefully assess whether the VNA titers of about 0.5 IU/mL are sufficiently protective when using the FAVN method as a standard VNA test.^39^ Dogs' sera having titers <0.5 IU/mL is considered as a failure in vaccination. The consequence of failure of vaccination is ineffable because the vaccine cannot provide the proof of adequate response against rabies vaccine. We did not test the same serum sample multiple times, so the reproducibility test was undone. Therefore, we should confirm with traditional FAVN and RFFIT when the titers by the FAVN technique are approximately 0.5 IU/mL. There may be changes in the antigenic conformation of G proteins between the challenge virus strains employed in the VNA experiment, which could account for the variances between RFFIT‐GFP and RFFIT. There haven't been many investigations into finding rabies antibodies in wild animals. Paquot et al.^40^ conducted the initial investigation on wild boars and roe deer in 1988 in Belgium. Cliquet et al.^41^ conducted a study where they found 40.2% (43/107) of the tested samples had an antibody titer greater than 0.5 IU/mL. When they employed the RFFIT method, 32.6% of the samples had a titer greater than 1 IU/mL. Vengušt et al.^42^ detected rabies antibodies in 2011 in 28% of samples through the ELISA method, where the threshold value was 0.5 IU/mL for comparison with FAVN and RFFIT. For determining the concentration of rabies‐neutralizing antibodies, both the FAVN test and the original RFFIT are regarded as reliable. However, these techniques have drawbacks, including the requirement for expensive, time‐consuming, and well‐trained staff due to the use of live RABV.^43^ Wasniewski et al.^44^ and Robardet et al.^45^ found discrepancies between the results because of sampling in several areas and habitat have an impact on immunity. More research is required to properly understand this observation. Dascalu et al.^34^ found an overall agreement of 65.66% between ELISA and FAVN. More than 86% of the results from the FAVN test on ELISA‐positive samples were associated. Only 36.59% of samples that underwent the FAVN test and tested negative for the ELISA test were also negative. Jakel et al.^46^ and Cliquet et al.^47^ observe a significant difference between the results found from vaccinated dogs, which indicates variation in immunogenicity. Additionally, Jakel et al.^46^ demonstrated that if dogs had received one or two vaccinations, dogs sampled up to 4 months after vaccination had a considerably higher chance of reaching the antibody response cut‐off than dogs examined at later time points independently. As the dog's isotype transitions from an IgM response to an IgG response as an immune response develops, Kennedy et al.^48^ argue that this lesser response may not be related to a lack of immunological protection as the total immunoglobulin measure may be proportionately more accounted for by IgG. This accords with research by Mansfield et al.^49^ and Kennedy et al.^48^ that showed a higher risk of low antibody titers with aging as well as in dogs under 1 year old compared to adults.

The strategic goal to eliminate canine rabies through dog vaccination programs by 2030 calls for the successful control of canine rabies because canines are largely to blame for the 60,000 annual human deaths globally.^50^ Rabies vaccinations should be licensed by appropriate authorities and adhere to any applicable national or international standards, according to the WHO and WOAH's recommendations. The clearance process for commercial vaccinations should also include independent verification by the regulatory agencies^21^, ^51^ Servat et al.^52^ tested 122 batches of rabies vaccine, and more than 99% of their samples passed the potency test. But they found NIH to be a problematic method, as only 42% of assays met the validation parameters even after repeat tests. Krämer et al.^53^ tried to validate the NIH test through the rabies vaccine, and they found a statistically significant potency that was reproducible and reliable. Daas et al.^54^ described the preparation of a biological RABV standard in which nine laboratories participated to develop a high‐potency standard. The Pitman Moore rabies strain was employed to make the vaccines by inactivating it with beta‐propiolactone. The batch of rabies vaccine was given a potency of 11 IU/vial based on the study's findings. Their obtained results showed good repeatability and reproducibility, with a 100% satisfactory potency value. Moreira et al.^55^ were able to estimate the rabies vaccination potency and get a clinically effective potency of 2.5 IU/SHD, which is satisfactory. The results reveal a strong correlation between the potencies identified by the NIH test and the SPT. The assay was able to tell potent vaccine lots apart from subpotent ones. Machado et al.^56^ evaluated the potency of the rabies vaccine for human use. They assessed reliability by CV% and confidence intervals, but the impact of the result was measured by regression, linearity, and parallelism. The result was statistically significant, and we were able to reduce eight mice per dilution. An alternative TRFIA and ELISA test method against the NIH test was constructed by Lin et al.^57^ Under ideal circumstances, the RABV glycoprotein's potency was good, and the approach yielded satisfactory effects when used on real samples. For potency values acquired from the current TRFIA and ELISA tests, the correlation coefficient was 0.912, and for those obtained from the current TRFIA and NIH tests, it was 0.903. According to these early findings, the TRFIA can replace ELISA with greater performance and may offer a promising alternative to the NIH test. The NIH has a variable method. The NIH test is an outdated procedure with high variability that hasn't undergone any recent improvements. Other drawbacks of this test include the fact that it is expensive, time‐consuming, necessitates a huge number of animals, and causes the animals great distress.^52^ The marketing of substandard or fake human vaccines, which results in the administration of ineffective vaccines, is a worldwide concern; thus, these results are nevertheless interesting. Due to this, both kids and adults are unintentionally living without protection from this disease that can be prevented.^50^

Although human rabies is completely curable with vaccination, it nevertheless continuously infects humans. The rabies vaccine is remarkably effective, risk‐free, and well‐tolerated. Developing countries like Bangladesh, where biopharmaceuticals are not common and their vaccination program is primarily dependent on imported vaccines. Manufacturing quality rabies vaccines is a blessing for a weak economy. If local manufacturing products are able to provide efficacious results, surely the country will save plenty on import costs. Our study will provide clear and reliable data on a newly developed product that ensures the safety of a large population from a deadly disease. This study may be helpful for the competent authorities in organizing and implementing mass parenteral vaccination. The national government should make sure that local vaccinations are potent enough to fulfill international standards, prohibiting the manufacturing of subpar vaccines. If rabies vaccinations are effective and of international quality, they should be used to help the world reach its 2030 goal of having no human rabies deaths caused by dogs.

## 5 CONCLUSION

It could be concluded that the dose–response relationship between the standard vaccine and the sample vaccine was satisfactory. The findings of the SRID testing of samples from 20 different batches of rabies vaccinations show that SRID techniques may be useful for the quantitative and qualitative testing of rabies vaccines as well as for the assay of the rabies glycoprotein antigen for research. The FAVN is also an effective method for quantifying serum RABV virus‐neutralizing antibodies and distinguishing between animals with and without sufficient immune responses. The NIH test has demonstrated the feasibility, reproducibility, and reliability of a limit test utilizing a relatively small number of animals in a serological assay as compared to the full vaccination challenge in vivo potency test. Our expanded program on immunization is pledged to eliminate rabies through mass vaccination programs, but import‐dependent vaccination programs require huge budgets. Our study implies the standard and quality of Bangladeshi‐manufactured rabies vaccines. Our local vaccine can provide sufficient immunogenicity against the RABV because of its high potency, which is able to protect against lethal rabies.

### 5.1 Limitations of our study

We had many limitations in our whole journey. For the FAVN test, we cannot manage the same species and pure breed dog. The Challenge Virus Standard for the NIH test was bought from ATCC. we don't have any facility to generate CSV. Though the ELISA method is more precise and accurate for in vitro potency tests, we pursued the SRID method, which is not a modern‐day test method. We tested only 20 batches because of the high price of mice and their maintenance.

### 5.2 Future perspective

Our study provides warranted proof of locally manufactured rabies vaccine, which will help to root out rabies not only in Bangladesh but also around the world, the goal set by WHO.

## AUTHOR CONTRIBUTIONS

Fahima Akter contributed to data curation, formal analysis, visualization, original draft. Md. Shamimuzzaman involved in conceptualization, data curation, funding acquisition, investigation, methodology, project administration, resources, software, supervision, validation and writing—review and editing.

## CONFLICT OF INTEREST STATEMENT

The authors declare no conflict of interest.

## ETHICS STATEMENT

This study was carried out in compliance with the ethical committee of the National Control Laboratory, Directorate General of Drug Administration, Ministry of Health and Family Welfare. ERC number is NC‐AL‐GNL‐ERC‐23‐002/F1‐01.

## Supporting information

Supporting information.
