# Supplementary material for: Potency, immunogenicity, and efficacy of rabies vaccine: In vitro and in vivo approach
Source: Immun Inflamm Dis. 2024 Feb 27;12(2):e1198. doi: 10.1002/iid3.1198 (PMC10898210; doi:10.1002/iid3.1198)
Supplement: Supplementary file 1 — Supporting information. [file IID3-12-e1198-s001.docx]

**Text and data not for publication**

**2.2.1 Reagent preparation**

0.1M Tris buffer was prepared by dissolving 6.05 g of tris-free base (MERCK, UK; Catalogue Number, 648315) and 0.575 g of sodium azide (Sigma-Aldrich; CAS 26628-22-8) in 500 mL of purified water, and pH was adjusted to 8.2. 0.9 g of sodium chloride (Scharlau lab; EC number: 231-598-3) was dissolved in 100 ml of purified water to make a 0.9% NaCl solution. Coomassie Brilliant Blue (Thermo Scientific; Catalog number: 20279) was the staining solution. 0.25 g of Coomassie Brilliant Blue was mixed in 100 ml of purified water, 125 mL of ethanol (DUKSAN Pure Chemicals Co., Ltd.; CAS No. 64-17-5), and 25 mL of acetic acid (DAEJUNG Chemicals; CAS No.: [64-19-7]). A de-staining solution was prepared in a 1000-ml volumetric flask by adding 70 mL of acetic acid, 100 mL of ethanol, 40 mL of glycerol, and the rest of the volume of water.

**Reference standard solution preparation:** The standard was reconstituted with 1.0 mL of WFI (water for injection). The standard was then serially diluted to 1:4, 1:8, 1:16, 1:32, and 1:64 of glycoprotein in 0.9% NaCl.

**Sample Solution:** The sample in the vial was reconstituted with 1.0 mL WFI and diluted to 1:4, 1:8, 1:16, 1:32, and 1:64 by 0.9% saline water.

**1% agarose gel preparation:** 0.6 g of agarose was added to 60 mL of 0.1 M tris buffer and boiled to make a clear solution. 600 µL rabies anti-serum was mixed at 50–55 °C. After being solidified, a 3-mm well was prepared for each dilution.

**Loading of samples and standards:** 4 µL (max) of the reference standard and sample was added to the wells. Ten minutes later, a foil-wrapped tray with clotted gel was transferred to the moisture chamber. After 24 h of incubation, the clotted gel was separated and transferred to a staining tray. After staining with staining solution, the clotted gel was taken out and washed with de-staining solution for 15 minutes.

**Calculation:** After drying, the diameter of the diffusion circle of the standard and sample was measured by an automatic zone reader. The zone area was calculated with calibrated Microsoft Excel. The concentration of antigen in the test sample was determined using the potency of the standard through the following formula:

Slope of the sample solution × Potency of the sample

Concentration of antigen =

Slope of the standard solution

**Calculation of the titers:** log10 (dilution 50) = -(x0-d/2+d ∑ri/ni)

Where: log10 (dilution 50) = log D_50_=logarithm of the dilution where 50% of the positive wells is found; that is, 50% of the fluorescence;

x0=-(log10 of the smallest dilution with all negative wells)

d=log10 of the dilution step

ri = number of negative wells and

ni= number of replicates.

For the sera: logD_50_ of the serum = [(number of negative wells/4) × log of dilution step – (log of dilution step/2) + log of the lowest dilution where there are four negative wells]

For the CVS virus: logD_50_ of the virus = [(number of positive wells/4 × log of dilution step – (log of dilution step/2) + log of the lowest dilution where there are four positive wells]

**Conversion of the titers**

The conversion of the logD_50_ titer of the serum in the IU/mL titer is performed by using the formula:

[(10^(serum logD50 value)^) × theoretical titer of positive reference serum 0.5 IU/mL]

Serum titer (IU/mL) =

(10^(theoretical logD50 of positive reference serum)^)

**2.4 National Institutes of Health-NIH test (Mice Challenge Test)**

The mouse challenge test was performed in accordance with The British Pharmacopeia and WHO (21), (22).

**2.4.1 Reagent preparation**

**Preparation of phosphate buffer saline (PBS) for virus:** 8.0 g NaCl (Scharlau lab; product code: SO0225), 0.2 g KCL (MERCK KGaA, Germany; Cat no.: 1049360500), 0.2 g KH_2_PO_4_ (MERCK KGaA, Germany; Cat no.: 1048731000) and 2.92 g Na_2_HPO_4_. 2H_2_O (Scharlau lab; product code: SO0339) was taken in a 1000 mL volumetric flask and volume filled to the marked level by purified water. The solution was dissolved and autoclaved at 121 °C and 15 psi for 15 min.

**Preparation of phosphate buffer saline (PBS) for vaccine:** 8.5 g NaCl, 2.04 g Na_2_HPO_4_ ((Scharlau lab; product code: SO0227)_,_ and 0.136 g KH_2_PO_4_ were dissolved in a 1000 mL volumetric flask containing purified water. The pH was adjusted to 7.2–8.0 and autoclaved.

**Preparation of PBS containing 2% Fetal Bovine Serum (FBS):** 2 mL FBS (Sigma-Aldrich, ES-009-B or ThermoFisher Gibco FBS- 16000044) was aseptically transferred and mixed with 50 mL PBS in a 100 mL volumetric flask. The volume was filled up to the mark with PBS for viruses.

**Preparation of rabies challenge virus suspension (CVS):** To prepare a 10% and 20% challenge virus suspension, an ampoule of instant thawed frozen virus (ATCC VR 959, CVS-11) was diluted with 2% horse serum diluent.

**Rabies virus titer detection:** The median effective dose (ED 50) was determined with 6-week-old mice. A tenfold serial dilution of CVS supernatant was prepared, and 0.03 mL of each CVS was intracerebrally inoculated in groups of 10 mice. Mice were observed for any deaths in the first five days between the 1st and 14th days. On the 14^th^ day, antibody titers were tested using the Reed-Muench method.

Log LD_50_/0.03 mL = Log_10_ of the virus dilution with mortality above 50% + (Proportional distance × Log_10_ of dilution factor)

Here,

Proportional distance (PD) = Mortality above 50%-50 / (Mortality above 50% - Mortality below 50%)

**Dilution of test and standard vaccines:** For the first immunization, test and standard vaccines were reconstituted with 1 mL WFI. Then, using PBS, three 5-fold dilutions (1:25, 1:125, and 1:625) of the standard and test vaccines were prepared.

**2.4.2 Immunization**

**First immunization:** After the completion of the quarantine period, 16 healthy Swiss Albino Mice (8 male and 8 female) were selected for each dilution. Each dilution was administered intraperitoneally to each mouse in 1.0 mL. Every mouse was kept in a separate case with proper labeling.

**Second immunization:** On the 7^th^ day after the first immunization, the second immunization was performed in the same manner.

**2.4.3 Challenge Test**

**Dilution of challenge virus standard (CVS):** On the 14^th^ day after the first immunization, CSV suspension was diluted to get the challenge virus with a titer of 50 LD_50_/0.03 mL.

**Challenge the mice with virus:** On the 14^th^ day after the first immunization, immunized mice and a virus suspension containing about 50 LD_50_/0.03 mL titer were taken. 0.03 mL of virus suspension was injected into each mouse. Mice were kept in an observation room.

**Dilution of challenge virus standard (CSV) for back titration:** Challenge virus with a titer of 50 LD_50_/0.03 mL is considered 10^0^. 0.2 mL of 10^0^ CVS suspension was mixed with 1.8 mL of PBS in a 15-mL falcon tube to make a 10^-1^ dilution. Then 10^-2^, 10^-3^, and 10^-4^ dilutions of the challenging virus for virulence titration (back titration) were performed.

**Back titration:** On the 14^th^ day of the first immunization, healthy mice weighing 11–15 g were taken. Then 0.03 mL of challenge virus from each dilution (10^-1^, 10^-2^, 10^-3^, and 10^-4^) was injected intracerebrally into each mouse.

**2.4.4 Observation**

Mice were observed daily for 14 days starting from the date of challenge, and deaths were recorded. The mice that died or manifested typical signs of encephalopathy on or after the 5^th^ day following the challenge were included.

**2.4.5 Reference Vaccine**

The Pitman Moore strain of RABV, generated in the Nil-2 cell line and inactivated using ß-propiolactone, served as the basis for the freeze-dried vaccine known as Biological Reference Preparation (BRP) batch No. 5, which is now being distributed by the European Directorate for the Quality of Medicines (EDQM). The assigned titer for this reference vaccine is 10 International Units (IU) per vial.

**2.4.6 Statistical analysis and validation of test results**

The all-or-none response is the foundation of the NIH potency test. As a result, a parallel-line model with at least three points for BRP No. 5 and the vaccination under consideration was employed. Statistical analysis was conducted with CombiStat software version 6.01.
